# Permutation Matrix Encryption Based Ultralightweight Secure RFID Scheme in Internet of Vehicles

**DOI:** 10.3390/s19010152

**Published:** 2019-01-04

**Authors:** Kai Fan, Junbin Kang, Shanshan Zhu, Hui Li, Yintang Yang

**Affiliations:** 1 State Key Laboratory of Integrated Service Networks, Xidian University, Xi’an 710071, China; kangjunbin0616@163.com (J.K.); zhushanshan@stu.xidian.edu.cn (S.Z.); lihui@mail.xidian.edu.cn (H.L.); 2 Key Laboratory of the Ministry of Education for Wide Band-Gap Semiconductor Materials and Devices, Xidian University, Xi’an 710071, China; ytyang@xidian.edu.cn

**Keywords:** RFID, internet of vehicles, matrix encryption, ultralightweight, security and privacy

## Abstract

Radio frequency identification (RFID) is a kind of non-contact automatic identification technology. The Internet of Vehicles (IoV) is a derivative of the Internet of Things (IoT), and RFID technology has become one of the key technologies of IoV. Due to the open wireless communication environment in RFID system, the RFID system is easy to be exposed to various malicious attacks, which may result in privacy disclosure. The provision of privacy protection for users is a prerequisite for the wide acceptance of the IoV. In this paper, we discuss the privacy problem of the RFID system in the IoV and present a lightweight RFID authentication scheme based on permutation matrix encryption, which can resist some typical attacks and ensure the user’s personal privacy and location privacy. The fast certification speed of the scheme and the low cost of the tag is in line with the high-speed certification requirement in the Internet of vehicles. In this thesis, the specific application scenarios of the proposed RFID authentication scheme in the IoV is also discussed.

## 1. Introduction

Radio Frequency Identification (RFID) is a technology that exchanges data through electromagnetic waves or electromagnetic fields, which enables the non-contact identification of specific objects [1]. Due to the convenience and low cost of RFID technology, RFID technology has been widely used in industrial automation, commercial automation, transportation management, etc. [2].

The Internet of Vehicles (IoV) is the future of the Vehicular Ad-Hoc Networks, interconnecting vehicles, vehicle Telematics, and connecting vehicles. Combining these technologies and learning from the Internet of Things (IoT), the IOV brings smartness into the vehicular environment, improving the vehicles’ intelligence and vehicles’ networking [3]. As a key member of the IoT, IoV will greatly change future life [4]. IoV is a brand-new network application, which is the application of Internet of Things technology in the intelligent transportation. RFID is the core foundation of the new generation of intelligent transportation system [5]. RFID can achieve fast and autonomous identification, without the intervention of manual operation. In addition, the RFID tag has small volume with strong anti-pollution ability and good durability, and the RFID reader has a large reading distance, so the RFID system can work normally in a harsh environment [6]. Because of the excellent performance of the RFID system, RFID technology has become one of the key technologies in the IoV. The RFID-based smart transportation systems make digital management of vehicles possible, including real-time tracking, intelligent traffic warning, vehicle operation monitoring, etc. [7].

However, the smart RFID-based transportation systems may disclose users’ privacy since the open wireless communication in RFID systems is vulnerable to many attacks. Users’ privacy in IoV mainly includes personal information and location information. The personal information refers to the user’s identity, vehicle license information, credit card information, and other personal information. Vehicle location information leakage can lead to malicious tracking [8]. In the early application of RFID technology in IoV, such as the ETC technology, limited by the restrained storage capacity and poor computing capability of RFID tags, the adopted RFID authentication schemes usually have a poor performance in security and privacy. With the development of RFID technology, the storage and computing capability of tags have been improved. Meanwhile, the emerging cloud computing can help improve the security and privacy protection capability of RFID authentication scheme. Lightweight RFID security authentication scheme has been applied in the IoV. Designing a privacy-protected RFID authentication scheme for the IoV environment will greatly promote the development of the IoV [9].

In this paper, on the premise of considering the application requirements of the Internet of vehicles environment and ensuring users’ privacy and security, a permutation matrix encryption based ultralightweight secure RFID scheme is designed for the IoV, which reduces tag costs and improves the authentication speed. The new scheme designed in this paper ensures users’ privacy in IoV and the scheme can resist common attacks such as replay attack, tag-tracking attack, and desynchronization attack. In addition, the proposed scheme can achieve high-speed authentication in the IoV, and the low cost of tags used in the scheme is conductive to large-scale application.

The rest of the paper is organized as follows: In Section 2, we introduced the background of the application of RFID technology in the Internet of vehicles and reviewed some existing protocols. Next, the paper presents the permutation matrix encryption based ultralightweight secure RFID scheme in IoV in Section 3. Section 4 shows the security and performance analysis of the new scheme. Finally, some conclusions of the proposed scheme and the discussion of the future work are introduced in Section 5.

## 2. Related Work

### 2.1. RFID Technology in IoV

RFID systems usually consist of three parts: Tag, Reader, and Back-end server. RFID tags are wireless transceivers equipped on objects for detection. Each tag has a certain amount of computing and storage capacity. RFID readers are wireless transceivers that transmit electromagnetic waves through radio frequency antennas to interact with tags. The reader communicates with the label through radio frequency and transfers data to the background server, realizing the information exchange between the background server and label. Back-end server usually refers to a database system with strong data processing capability and storage capacity, which stores and manages the data related to tags. It can judge and verify the information from the tags [10]. The authentication cost also needs to be taken into account for the practical application [11]. One of the most important factors is tag’s computing overhead, which is often measured by the number of logic gates. Tags have very limited resources, about 5000 to 10,000 logic gates in total, of which only 2000 to 3000 logic gates can be used for encryption and authentication. In addition, the resource-constrained tags can only store a few hundred bits of data. The cost of low-cost tags mainly depends on the encryption and authentication method of the protocol [12].

As a kind of autonomous non-contact identification technology, RFID plays an important role in the construction of vehicle identification systems. Its efficient identification without manual intervention is suitable for high-speed driving scenarios of vehicles. It is one of the indispensable communication methods in the IoV [13].

RFID technology has many applications in the Internet of vehicles. The IoV built with RFID technology can realize the function of electronic license plates, which can cooperate with traditional license plates to achieve counterfeit license plates identification, traffic flow monitoring, Electronic Toll Collection (ETC), intelligent parking management, traffic safety warning, multi-path identification, and other functions [14].

### 2.2. Requirements for RFID Authentication Schemes in IoV

The user’s privacy in the IoV includes the user’s personal privacy, and the user’s position privacy when driving the vehicle, either the leakage of the user’s personal privacy or the leakage of the vehicle’s location privacy will cause trouble to the user. The RFID authentication scheme applicable to the IoV environment should have both the ability to authenticate quickly and the ability to protect privacy [15].

### 2.3. Karthikeyan-Nesterenko’s Protocol Based on Matrix Operation

Karthikeyan-Nesterenko’s protocol realizes information encryption and authentication based on simple XOR operation and matrix operation. The key shared between the background server and the tag in the protocol is dynamic, and the key is updated after every authentication. However, the protocol cannot resist Denial of Service attacks (DOS), replay attacks, and tag tracking attacks. In the protocol, the tag does not authenticate the message received from the reader when updating the key. As a result, an attacker can impersonate as a legitimate reader with an old message that was intercepted before the current authentication, and the tag will authenticate the fake message successfully and update the key with the fake message when tag receives the fake message. So, the legitimate reader and the tag cannot authenticate each other anymore since the key is wrongly updated. The protocol is also unable to resist replay attack so that the attacker can track the tag through replay attack, thereby causing the privacy leakage. Another problem of the protocol is that when the backend server confirms the validity of the tag it needs to search the database to find a match, and the search process will cost a lot of time when the data is large [16].

Based on Karthikeyan-Nesterenko’s protocol, the new scheme proposed in this paper consumes less computing resources and meets the security requirements of IoV. The new protocol improves the privacy flaws in Karthikeyan-Nesterenko’s protocol. We use Unix timestamp which is generated by the reader to resist replay attack and tag tracking attack. Moreover, the timestamp replaces the random number which is generated by the tag in Karthikeyan-Nesterenko’s protocol. The usage of Unix timestamp in our scheme contributes to accelerate the authentication and reduce the computation overhead of the tag. Meanwhile, in order to meet the requirements of the fast authentication in the IoV, we have improved the way by which the back-end server authenticates tags. In our scheme, the back-end server does not need to search data in the database to identify tags and this improvement makes our scheme is suitable for the situation where large amounts of data are stored in the database. Considering the high speed of vehicles during authentication process, we reduce the communication times during a whole authentication process. In terms of encryption mechanism, we use permutation matrix to encrypt the data that will reduce the computing and storage overhead of the tag.

There are other articles discussed Karthikeyan-Nesterenko’s protocol and proposed their improved schemes, such as [17,18,19]. They have pointed out the shortcomings of Karthikeyan-Nesterenko’s protocol, but they do not use matrix to encrypt the data. We encrypt data with permutation matrix based on Karthikeyan-Nesterenko’s protocol in our protocol. Compared with other protocols, our scheme is more suitable for IoV.

### 2.4. Kim’s Protocol and Chien’s Protocol

Kim’s protocol is a hash-based key exchange protocol which protects exchanged messages via hash functions. Kim’s protocol saves old and new secret values in the back-end server that makes it resist most of the attacks. But Kim’s protocol is vulnerable to replay attacks on readers or tags. The adversary can attack the tag or reader successfully with the probability “1/4” [20].

SASI is an ultra-lightweight RFID authentication which proposed by Chien et al. [21]. The low-cost operations bitwise XOR and Rot are used in SASI to reduce computing overhead. SASI can resist the replay attack and ensure synchronization during the session. But SASI is unable to resist the tag-tracking attacks and ensure the tag anonymity. The SASI could not protect the user’s location privacy if it is applied to IoV.

## 3. Permutation Matrix Encryption Based Ultralightweight Secure RFID Scheme for IoV

### 3.1. Permutation Matrix

A permutation matrix is a matrix obtained by permuting the rows of an identity matrix according to some permutation of the numbers “1”. Every row and column therefore contains precisely a single 1 with 0 s everywhere else, and every permutation corresponds to a unique permutation matrix. The product of the permutation matrix and its transposed matrix is the identity matrix. So we can use the permutation matrix as a key to encrypt information and decrypt the information with the transposed matrix of the permutation matrix, since there is only one "1" in each row and column of the permutation matrix. When we decrypt the data encrypted by permutation matrix, we can obtain the decrypted data by directly transforming the position of “0” or “1” in the data through the position of “1” in the permutation matrix. This can simplify the circuit in the tag, and reduce the number of logic gates in the tag and the time spend on calculation.

### 3.2. Permutation Matrix Encryption Based Ultralightweight Secure RFID Scheme

When designing the protocol, we assume that the data transmission between the background server and the reader of the system is secure. The protocol proposed in this paper focuses on the authentication and information security between reader and tag. The system notations are presented in Table 1.

As indicated in Table 1, $T_{1}$ represents the current time that the reader receives from the Internet when the reader makes an authentication request to the tag; $T_{0}$ represents the time when the reader is successfully identified by the tag in the last session. The back-end server use the secret key *S* shared between it and the tag to identify and authenticate the tag. *R* represents the random number generated by the reader each time when the reader sends query request to the tag. $M_{1}$, $M_{2}$ are two 128×128 permutation matrices stored in the tag for both decryption and encryption. $M_{1}^{-1}$, $M_{2}^{-1}$ are two 128×128 transposed matrix of $M_{1}$ and $M_{2}$ stored in the reader.

**Figure 1:**
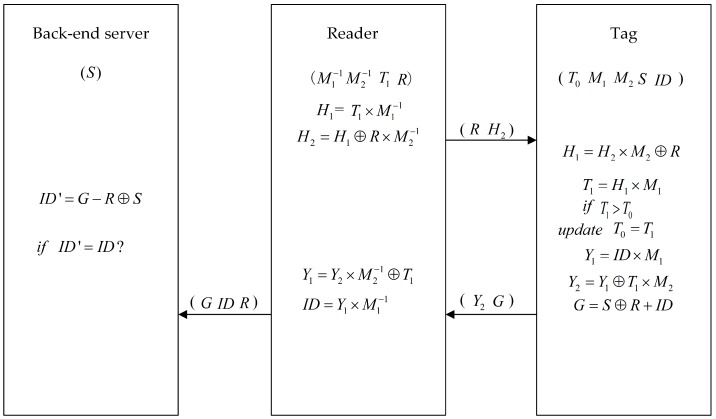
Proposed scheme.

Our scheme consists of two phases, including the initialization phase and the authentication phase, which is shown in Figure 1. The details of the protocol are as follows.

#### 3.2.1. Initialization Phase

In the initialization phase, the back-end server shares the secret key *S* with each legitimate tag. The legitimate reader and tag store the corresponding permutation matrix respectively. The reader is connected with the Internet to get a real-time Unix timestamp.

#### 3.2.2. Authentication Phase

1. Reader → Tag: *R*, $H_{2}$
  The reader generates random number *R* and encodes the current network time as $T_{1}$. The reader encrypts $T_{1}$ with permutation matrix $M_{1}^{-1}$, $M_{2}^{-1}$: $H_{1}=T_{1}\times M_{1}^{-1}$, $H_{2}=H_{1}⊕R\times M_{2}^{-1}$. Then the reader sends *R* and $H_{2}$ to tag as a challenge.
2. Tag → Reader: $Y_{2}$, *G*
  After receiving *R* and $H_{2}$, the tag uses *R* and $M_{1}$, $M_{2}$ to decode $H_{2}$ to get $T_{1}$. The tag will compare $T_{1}$ with $T_{0}$ stored in tag. Only when the last 64 bits of $T_{1}$ are greater than $T_{0}$ no more than 48 h that the reader is authenticated. If the reader is authenticated, the tag updates the value of $T_{0}$ with $T_{1}$ and uses the updated $T_{0}$ to compare with next $T_{1}$ in next session. Then the tag encrypts the ID in two ways. In the first way: $Y_{1}=ID\times M_{1}$, $Y_{2}=Y_{1}⊕T_{1}\times M_{2}$. $Y_{2}$ is the the encrypted ID with permutation matrix $M_{1}$, $M_{2}$, and $T_{1}$ by the tag. G=S⊕R+ID, in this way, ID is encrypted with the secret key *S*.
3. Reader → Back-end server: ID, *R*, *G*
  The reader decrypts ID from $Y_{2}$ after receiving $Y_{2}$, *G*. Reader sends *G*, ID, *R* to the back-end server. Back-end server calculates $ID^{\prime }=G-S⊕R$. The back-end server compares $ID^{\prime }$ with ID. If $ID^{\prime }$ is same with ID, the tag is legitimate. Therefore, mutual authentication is achieved.

### 3.3. Protocol Implementation Details

#### 3.3.1. Data Encoding

In the proposed protocol, $T_{1}$, $T_{0}$, *R*, and ID are all 128-bit binary numbers. The secret key *S* shared by the back-end server and tag and the permutation matrix used for encryption and decryption are distributed by trusted third parties, which is the basis of information confidentiality of the protocol. The secret key *S* and ID should be coded with redundant information to ensure both the number of “0” and “1” are 64 bits, to improve the confidentiality of protocol. The same rules should be followed when coding $T_{1}$ to prevent attackers from cracking the data using exhaustive method. $T_{1}$ is encoded in a way that the first 64 bits are randomly filled by the reader to make the number of “1” and “0” are both 64 in $T_{1}$. The latter 64 bits in $T_{1}$ represents the Unix timestamp which is the number of seconds that have elapsed since 00:00:00 Coordinated Universal Time (UTC), Thursday, 1 January 1970.

#### 3.3.2. The Tag

The tag adopted in the scheme is active tag which is powered by vehicle to meet the requirements of launch distance in the IoV. The tag stores $T_{0}$ in the registers and the registers will lose the value of stored $T_{0}$ after the power is off. So the tag will initialize $T_{0}$ to the current time each time when the vehicle starts.

In our scheme, both the encryption and decryption of the data in the tag are completed by multiplying $M_{1}$ and $M_{2}$. The same matrix multiplication circuit can be used to encrypt and decrypt in the tag to reduce the cost of tags.

#### 3.3.3. The Role of *R* and $T_{1}$

The operation $H_{1}⊕R$ is designed to prevent the attacker from tracking the vehicle through a linear increase in $T_{1}$. Similarly, operation $Y_{1}⊕T_{1}$ and S⊕R is also used to update $Y_{2}$ and *G* in different sessions to prevent the attacker from tracking the vehicle. $T_{1}$ also acts as a shared secret key between the reader and the tag. The tag computes $Y_{1}⊕T_{1}$ to encrypt ID. The reader can get the correct ID with the correct $T_{1}$.

## 4. Analysis and Evaluation

The security and privacy analysis and performance evaluation of the proposed scheme are given in this section. In addition, the security proof of this protocol is described by BAN logic proof.

### 4.1. Security and Privacy Analysis

- Tag anonymity: Tag anonymity is the basis of the RFID system to protect users’ privacy in the IoV. In the proposed protocol, the secret data ID is encrypted by the 128×128 permutation matrices and the number of “1” and “0” are both 64 in $T_{1}$ and ID. Even if the attacker gets the $H_{2}$ or *G*, at least $C_{128}^{64}\times C_{128}^{64}$ times operation needed to obtain the secret data ID or $T_{1}$. According to the current computing power of computers, it is deemed that encrypted data cannot be obtained by brute force. Thus, the secret data is safe in our scheme and the tag anonymity is ensured.
- Resistance to tag-tracking attacks: The tag-tracking attack refers to the attacker can determine a certain tag by eavesdropping the data transmitted during communication. So the tag can be tracked. In our scheme, both $T_{1}$ and *R* updated in every session, so the data $Y_{2}$ and *G* that tag sends to reader differs in every session. It is difficult for the attackers to find the correlations of data in two sequential sessions. The proposed scheme can resist tag-tracking attacks.
- Mutual authentication: In the protocol, the permutation matrix in the tag and the reader is secret. Only the legitimate tag and the legitimate reader have the corresponding encryption and decryption matrix. The secret key *S* shared by the background server and labels is also distributed by trusted third parties and is confidential. The purpose of the tag decrypting $H_{2}$ to get $T_{1}$ and comparing $T_{1}$ with $T_{0}$ is identifying the legitimate reader. Only the legitimate reader can be authenticated successfully. Also, the reader can only obtain the correct ID after the tag encrypts it with the corresponding permutation matrix. When the ID obtained by the reader is equal to the $ID^{\prime }$ decrypted by the back-end server using the secret key *S*, the back-end server authenticates the tag successfully. Thus, the mutual authentication is ensured in the new scheme.
- Resistance to replay attacks: Replay attack refers to that an attacker, after stealing a valid message between a tag and a reader transmitted in the previous session, sends the obtained message to the original receiver for authentication. In our scheme, the tag stores the $T_{0}$ which is the time of reader authenticated successfully in last session. If the attacker sends the old (*R*,$H_{2}$) to the tag, $T_{1}$ that the tag decrypts from the old (*R*,$H_{2}$) will not greater than $T_{0}$ and the tag will identify the attacker to be illegal. In the same way, if the attacker sends the old ($Y_{2}$,*G*) to the reader, wrong ID will be decrypted and the back-end server will identify the attacker as an illegal tag. In the application scenarios, the time interval between two successful authentication sessions is much longer than one second. In our scheme, when the tag compared $T_{1}$ and $T_{0}$, only when $T_{1}$ is bigger than $T_{0}$ will the reader be authenticated. Therefore, the attacks which are carried out immediately (delta t < 1 s) will not be successful. Our scheme can resist replay attacks.
- Resistance to de-synchronization: The permutation matrix between the tag and the reader and the key *S* shared between the tag and the server do not need to be updated. The updated time $T_{1}$ and the random number *R* in each session are generated by the reader, and the reader will update $T_{0}$ only after it successfully authenticates the tag. The tag and the server will not mutually authenticated unsuccessfully because of one authentication failure, so the proposed protocol can effectively resist the de-synchronization attack.

A comparison of our proposed scheme with recent schemes is listed in Table 2. From the comparison of the security in Table 2, it can be seen that the protocols listed in the table have more or less security performance defects. The protocol designed in this paper has better security. It can effectively resist various common attacks. In addition, the proposed scheme can prevent the leakage of users’ privacy in the IoV effectively. Thus, the proposed scheme meets the design objectives and requirements of the protocol.

### 4.2. Performance Evaluation

**Figure 2:**
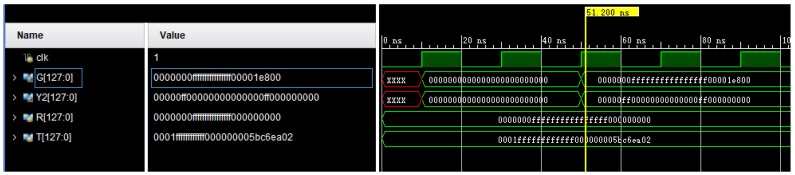
Behavioral simulation.

**Figure 3:**
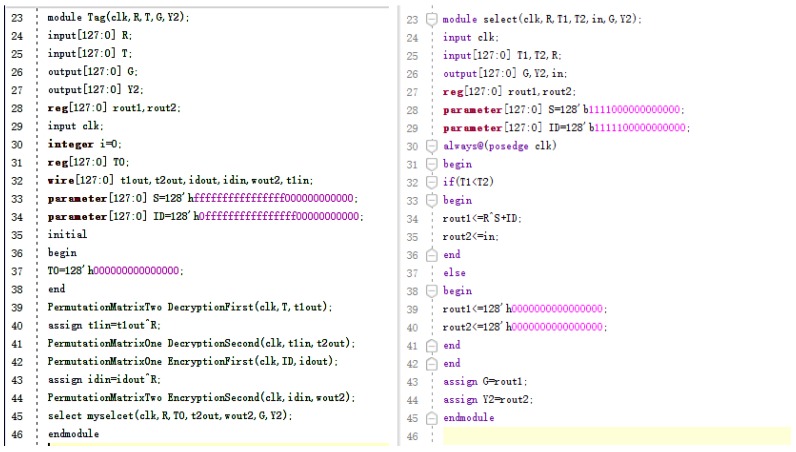
Using Verilog HDL to design label circuit.

We conducted FPGA based instantiation and synthesis in Vivado 2017.4 environment for Virtex-7 FPGAs with 128-bit data input. The simulation results are shown in Figure 2 and the part of Verilog HDL is shown in Figure 3.

We compared resource utilization of our protocol and other ultralightweight RFID authentication protocols in Table 3. Umar Mujahid’s protocol [22] simulated with 96-bit data input and Sadaiyappan’s protocol [23] simulated with 32-bit data.

We also compare the performance of other authentication protocol with the new scheme in Table 4. In Table 4, “⊕” is the XOR operation. “+” is the additive operation. “∨” is the OR operation. “Rot” is the cascade operation. “×” is the multiplication with permutation matrix operation, “Hash” is the displacement operation, and “PRNG ” is the hash operation. “PRNG” is a preset operation for EPC Class-1 Gen-2 tags, denoted in the EPC Class-1 Gen-2 Standard. As we can see, the proposed scheme just involves simple operations “⊕”, “+” and operation “×”. “⊕” and “+” are simple bitwise operations and both of them are low-cost. When the tag stores the permutation matrix, the tag only needs to store the position of "1" in each column. In the circuit where 128-bit data is multiplied by the replacement matrix, the result after replacement can be obtained according to the position of “1” in the replacement matrix stored in the tag, without a lot of calculation. In our scheme, tags do not require “Hash” operation and “PRNG” operations. Only simple bitwise operations, lookup operations, and comparison operations are required. The cost of the tag in the new scheme is presented in Table 3. “LUT” refers to “Look-Up-Table” and it is the structure of the smallest unit of the FPGA. It can be seen from the table that the tags we used in our scheme require few FPGA resources, and the production cost of the tags is low, which is beneficial to the large-scale application in the IoV.

In addition, there is only one communication between the tag and reader during the whole authentication process in our scheme. Compared to other protocols that require multiple communications between the tag and the reader during the authentication process, our scheme is more applicable to the mutual authentication between readers and high-speed vehicles in the context of IoV.

### 4.3. BAN Logic Proof

In 1989, Burrows, Abadi, and Needham presented a logic based on knowledge and belief, the main idea of which was to introduce new beliefs from known beliefs based on subjective beliefs. In this section, we use the BAN logic to formally analyze the proposed protocol.

Before BAN logical proof process, we briefly introduce its symbols and rules that we will use.

Symbols:

- P|≡X: The entity *P* believes *X* is true.
- P◃X: The entity *P* receives the message containing *X*, which means a certain entity *Q* sends a message containing *X* to *P*.
- P|∼X: The entity *P* has sent out message containing *X*.
- #(X): *X* is fresh.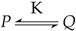: *K* is the shared secret of entity *P* and entity *Q*, and other entities do not know *K* except *P* and *Q*.
- P→Q: *P* sends messages to *Q*.
- ⊢: A meta-linguistic symbol, which means that the conclusion is a drawn from premise.

Rules:

- Message-meaning rules 
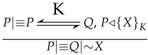
: If *P* believes that *K* is the shared secret between *P* and *Q*, and receives the message ${\left\{X\right\}}_{K}$, then *P* believes that *Q* has sent message *X*.
- Freshness rule $\frac{P|\equiv \#(X)}{P|\equiv \#(X,Y)}$: If a part of the message *X* is fresh, the message (X,Y) is fresh.

In our BAN logical proof, T denotes the tag; R denotes the reader; BS denotes the back-end server. Proof process is divided into five parts:

#### 4.3.1. Protocol Description

1. $Re\to T:\{R,\;T_{1}∼M_{1}^{-1}⊕R∼M_{2}^{-1}\}$
2. $T\to Re:\{S⊕R+ID,\;ID∼M_{1}⊕T_{1}∼M_{2}\}$
3. Re→BS:{R,S⊕R+ID,ID}

#### 4.3.2. Protocol Idealization

1. $Re\to T:\left\{R,\;{\left\{T_{1},R\right\}}_{M_{1}^{-1},M_{2}^{-1}}\right\}$
2. $T\to Re:\left\{{\left\{R,ID\right\}}_{S},\;{\left\{ID,T_{1}\right\}}_{M_{1},M_{2}}\right\}$
3. $Re\to BS:\left\{R,\;{\left\{R,ID\right\}}_{S},\;ID\right\}$

#### 4.3.3. Initial Assumptions

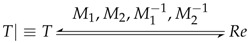

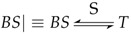

3. BS|≡#(Re)

#### 4.3.4. Proving Goals

1. T|≡Re
2. BS|≡T|∼#{R,ID}

#### 4.3.5. Proof Process

From BAN logic message-meaning rules 
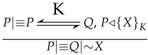
, Initial Assumptions 1 and Protocol Idealization 1, we can get:

(1) $$T\to Re:\left\{{\left\{R,ID\right\}}_{S},\;{\left\{ID,T_{1}\right\}}_{M_{1},M_{2}}\right\},\;T◃\left\{R,\;{\left\{T_{1},R\right\}}_{M_{1}^{-1},M_{2}^{-1}}\right\}⊢T\left|\equiv Re\right|∼T_{1}$$

The tag can determine the correctness of $T_{1}$ by the time of the vehicle. If $T_{1}$ meets the requirements, the tag authenticates the reader. That is Proving Goals 1.

From BAN logic message-meaning rules 
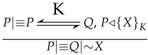
, Initial Assumptions 2 and Protocol Idealization 3, we can get: (2)
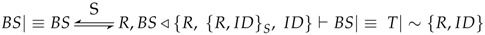


From BAN logic freshness rule $\frac{P|\equiv \#(X)}{P|\equiv \#(X,Y)}$ and Initial Assumptions 3, we can get:

(3) BS|≡#(Re)⊢BS|≡#{R,ID}

Therefore, we get BS|≡T|∼#{R,ID}. Proving Goals 2 is proved.

## 5. Discussion and Conclusions

With the rapid development and application of Internet of Things technology, IoV technology as a vertical application of the Internet of Things has great development prospects. Although RFID has found wide applications in daily life, the security problems and privacy problems caused by its open wireless communication environment still hinder its further development. In the application of the IoV technology, users pay more attention to privacy security. Designing a secure RFID authentication protocol suitable for the IoV is a challenging research topic. On the basis of studying the security issues and privacy requirements of IoV technology, this paper designs an RFID authentication protocol that can resist common attacks and ensure the privacy of users. The protocol encrypts and authenticates related information based on the permutation matrix. The current time and random numbers involved in the update phase make the proposed scheme resistant against replay attack and ensure users’ data privacy and location privacy. The protocol authentication process is simple and high-speed. The authentication process does not require the back-end server to do a lot of data searching, and the tag ID can be obtained after successful authentication, which is applicable to the situation of massive data stored in the back-end server in the Internet of vehicles. In addition, the simulation results show that the tag circuit uses fewer gates. Thus, less computing resources are required in the authentication process, which is suitable for large-scale applications.

The protocol designed in this paper can be applied to traffic statistics, toll collection systems of roads and bridges, vehicle access management systems, and so on due to its fast certification speed, strong information confidentiality, and non-repudiation after the tag is authenticated successfully. According to the positioning function of the RFID system, the protocol can also be applied to location-based IoV services such as navigation services.

The security of the protocol can be further improved. In the protocol designed in this paper, the replacement matrix used for encryption and the secret key shared by the tag and the background server are both fixed, which increases the possibility and harmfulness of information leakage. The protocol can be improved for this weakness and the permutation matrix and shared secret key could be updated after every authentication under the premise of ensuring protocol security, so as to reduce the possibility of the permutation matrix and key being cracked.

In the future, the protocol can be improved to reduce the tag cost. For example, the length of data can be reduced by optimizing the encryption method on the premise of ensuring the protocol security, so as to reduce the tag computational costs.

## Figures and Tables

**Table 1:** List of notations used.

Notation	Description
$T_{1}$	The time the reader makes a request to the tag
$T_{0}$	Last successful authentication time stored in the tag
*R*	Random number generated by the reader
*S*	The secret key shared between back-end server and tag
ID	Unique identification information of the specific tag
$M_{1}M_{2}$	Permutation matrix used for encryption and decryption in tag
$M_{1}^{-1}M_{2}^{-1}$	Permutation matrix used for encryption and decryption in reader
$H_{1}H_{2}$	The encrypted $T_{1}$ generated by reader
$Y_{1}Y_{2}$	The encrypted ID generated by the tag
*G*	The result of ID encrypted by *S*

**Table 2:** Security comparison.

	Karthikeyan Protocol	Kim Protocol	Chien Protocol	Proposed Protocol
Tag anonymity	Y	Y	N	Y
Tag-tracking arrack	N	Y	Y	Y
Mutual authentication	Y	Y	Y	Y
Replay attack	N	N	Y	Y
Desynchronization	N	Y	Y	Y

**Table 3:** Resource the tag used in tag.

Resource	Umar Mujahid’s Protocol	Sadaiyappan’s Protocol	Proposed Protocol
Number of Slice Registers	879	32	384
Number of Slice LUTs	1126	426	197

**Table 4:** Computation cost comparison.

Protocols	Tag Cost
Karthikeyan protocol	⊕,Matrixmultiplication
Kim protocol	⊕,||,Hash,PRNG
Chien protocol	⊕,+,∨,Rot
Proposed protocol	⊕,+,×

## References

[1] Zhang B., Ma X.X. (2013). Design and Analysis of a Lightweight Mutual Authentication Protocol for RFID. J. Univ. Electron. Sci. Technol. China.

[2] Liu Y.L., Qin X.L., Zhao X.J., Hao G.S., Dong Y.Q. (2015). Lightweight RFID Authentication Protocol Based on Digital Signature. Comput. Sci..

[3] Mendiboure L., Chalouf M.A. Towards a Blockchain-Based SD-IoV for Applications Authentication and Trust Management. Proceedings of the 5th International Conference.

[4] Sun X.H. (2013). Key Technology and Its Application of IoV. Commun. Technol..

[5] Nan C.L., Liu S.C., Zhou S.J., Zhao X.N. (2013). RFID Model for the Internet of Vehicles. Comput. Syst. Appl..

[6] Yan Z.G., Liu Z.C. (2011). The Preliminary Study on the Test Platform for the Internet of Vehicles. Mechatronics.

[7] Han W., Li L. (2013). Analysis based on vehicle networking application and construction of RFID technology. Electron. Test.

[8] Liu Y.L. (2014). RFID Secure Authentication Protocol for Privacy-Preserving. Ph.D. Thesis.

[9] Ren Z.G., Gao Y.B. Design of Electronic Toll Collection System in Expressway Based on RFID. Proceedings of the International Conference on Environmental Science and Information Application Technology.

[10] Ahmed M., Zouheir L., Mohamed S., Mostafa B. A novel mutual authentication scheme for low-cost RFID systems. Proceedings of the International Conference on Wireless Networks and Mobile Communications.

[11] Fan K., Jiang W. (2018). Lightweight RFID Protocol for Medical Privacy Protection in IoT. IEEE Trans. Ind. Inform..

[12] Chen X.Z. (2013). Research on Car-network Technology. Comput. Knowl. Technol..

[13] Fan K., Wang W. (2018). Secure ultra-lightweight RFID mutual authentication protocol based on transparent computing for IoV. Peer-to-Peer Netw. Appl..

[14] Musfiq R., Raghav S., Srinivas S. Lightweight protocol for anonymity and mutual authentication in RFID systems. Proceedings of the 12th Annual IEEE Consumer Communications and Networking Conference.

[15] Mei Y. (2014). Research on the Privacy Preservation for VANET. Ph.D. Thesis.

[16] Ahmed M., Mohamed S., Zouheir L., Mostafa B. Security analysis of low cost RFID systems. Proceedings of the 5th Workshop on Codes, Cryptography and Communication Systems.

[17] Chen Y.H. (2007). Mutual authentication protocol for RFID conforming to EPC Class 1 Generation 2 standards. Comput. Stand. Interfaces.

[18] Chien C.L. (2009). Conformation of EPC Class 1 Generation 2 standards RFID system with mutual authentication and privacy protection. Eng. Appl. Artif. Intell..

[19] Yu C.H. An Ultralightweight Mutual Authentication Protocol for EPC C1G2 RFID Tags. Proceedings of the Fifth International Symposium on Parallel Architectures, Algorithms and Programming.

[20] Behzad A., Karim B. Securing Key Exchange and Key Agreement Security Schemes for RFID Passive Tags. Proceedings of the 24th Iranian Conference on Electrical Engineering.

[21] Chien H.Y. (2007). SASI: A New Ultralightweight RFID Authentication Protocol Providing Strong Authentication and Strong Integrity. IEEE Trans. Dependable Secure Comput..

[22] Sadaiyappan T., Manoj K.K. (2014). FPGA Implementation of Mutual Authentication Protocol Using Modular Arithmetic. Int. J. Comput. Sci. Mob. Comput..

[23] Sadaiyappan T., Manoj K.K. (2016). A New Ultralightweight RFID Mutual Authentication Protocol: SASI Using Recursive Hash. Int. J. Distrib. Sens. Netw..

